# Embryonal tumor with abundant neuropil and true rosettes (ETANTR), ependymoblastoma, and medulloepithelioma share molecular similarity and comprise a single clinicopathological entity

**DOI:** 10.1007/s00401-013-1228-0

**Published:** 2013-12-14

**Authors:** Andrey Korshunov, Dominik Sturm, Marina Ryzhova, Volker Hovestadt, Marco Gessi, David T. W. Jones, Marc Remke, Paul Northcott, Arie Perry, Daniel Picard, Marc Rosenblum, Manila Antonelli, Eleonora Aronica, Ulrich Schüller, Martin Hasselblatt, Adelheid Woehrer, Olga Zheludkova, Ella Kumirova, Stephanie Puget, Michael D. Taylor, Felice Giangaspero, V. Peter Collins, Andreas von Deimling, Peter Lichter, Annie Huang, Torsten Pietsch, Stefan M. Pfister, Marcel Kool

**Affiliations:** 1 Clinical Cooperation Unit Neuropathology, German Cancer Research Center (DKFZ), Heidelberg, Germany; 2 Department of Neuropathology, Heidelberg University Hospital, Heidelberg, Germany; 3 Division of Pediatric Neurooncology, German Cancer Research Center (DKFZ), Im Neuenheimer Feld 280, 69120 Heidelberg, Germany; 4 Department of Pediatric Hematology and Oncology, Heidelberg University Hospital, Heidelberg, Germany; 5 Department of Neuropathology, NN Burdenko Neurosurgical Institute, Moscow, Russia; 6 Division of Molecular Genetics, German Cancer Research Center (DKFZ), Heidelberg, Germany; 7 Department of Neuropathology, University of Bonn, Bonn, Germany; 8 Arthur and Sonia Labatt Brain Tumor Research Centre, Hospital for Sick Children, University of Toronto, Toronto, Canada; 9 Departments of Pathology and Neurological Surgery, Brain Tumor Research Center, University of California, San Francisco, USA; 10 Department of Pathology, Memorial Sloan Kettering Cancer Center, New York, USA; 11 Department of Radiological, Oncological and Anatomic Pathology Sciences, Università Sapienza, Rome, Italy; 12 IRCCS Neuromed, Pozzilli, Italy; 13 Department of Neuropathology, Academic Medical Center, Amsterdam, The Netherlands; 14 Center of Neuropathology, Ludwig-Maximilians University, Munich, Germany; 15 Institute of Neuropathology, University Hospital Münster, Münster, Germany; 16 Institute of Neurology, Medical University of Vienna, Vienna, Austria; 17 Department of Pediatric Neurooncology, Dmitry Rogachev Federal Research Center of Pediatric Hematology, Oncology and Immunology, Moscow, Russia; 18 Department of Pediatric Neurosurgery, Necker Hospital, Université Paris Descartes, Sorbonne Paris Cité, Paris, France; 19 Department of Pathology, University of Cambridge, Cambridge, UK

## Abstract

Three histological variants are known within the family of embryonal rosette-forming neuroepithelial brain tumors. These include embryonal tumor with abundant neuropil and true rosettes (ETANTR), ependymoblastoma (EBL), and medulloepithelioma (MEPL). In this study, we performed a comprehensive clinical, pathological, and molecular analysis of 97 cases of these rare brain neoplasms, including genome-wide DNA methylation and copy number profiling of 41 tumors. We identified uniform molecular signatures in all tumors irrespective of histological patterns, indicating that ETANTR, EBL, and MEPL comprise a single biological entity. As such, future WHO classification schemes should consider lumping these variants into a single diagnostic category, such as embryonal tumor with multilayered rosettes (ETMR). We recommend combined LIN28A immunohistochemistry and FISH analysis of the 19q13.42 locus for molecular diagnosis of this tumor category. Recognition of this distinct pediatric brain tumor entity based on the fact that the three histological variants are molecularly and clinically uniform will help to distinguish ETMR from other embryonal CNS tumors and to better understand the biology of these highly aggressive and therapy-resistant pediatric CNS malignancies, possibly leading to alternate treatment strategies.

## Introduction

According to the 2007 WHO classification of tumors of the central nervous system (CNS), CNS primitive neuroectodermal tumors (PNETs) can be further subdivided into CNS neuroblastoma/ganglioneuroblastoma, medulloepithelioma (MEPL), and ependymoblastoma (EBL) [18]. In addition, “embryonal tumor with abundant neuropil and true rosettes” (ETANTR) has been discussed as a possibly unique variant of CNS PNET [1, 2, 4, 6, 8, 10, 11, 19].

CNS neuroblastomas histologically and molecularly resemble subsets of medulloblastomas and peripheral neuroblastomas [18]. They are characterized by the presence of Homer Wright (neuroblastic) rosettes, foci of neurocytic and/or ganglion cell maturation, intense synaptophysin expression, and *MYC/MYCN* amplifications in almost 50 % of cases [3, 18]. On the other hand, ETANTR, EBL, and MEPL are rare neoplasms characterized by the presence of similar histological patterns, namely multilayered and pseudo-stratified rosette-forming structures of variable shape and size. Both EBL and ETANTR include the so-called “ependymoblastic rosettes” harboring well-formed central round or slit-like lumina in the absence of an outer membrane [4, 6, 11, 12, 14, 18]. MEPL is histologically characterized by papillary and tubular structures surrounded by an external limiting membrane, reminiscent of the developing neural tube [4, 18]. These structures are sometimes also referred to as “medulloepithelial” rosettes. Moreover, some MEPL have also been reported to display ependymoblastic rosettes [18]. These three variants of embryonal CNS tumors show a clinically uniform behavior, in that they predominantly affect infants under the age of 4 years and are associated with a highly aggressive course with reported survival times up to 24–36 months, but typically averaging 12 months [1, 5, 9, 11, 15, 23].

Applying FISH analysis, we previously found amplifications at 19q13.42 involving the *C19MC* cluster in 93 % of tumors diagnosed either as ETANTR, EBL, or MEPL with ETANTR features, but not in any other pediatric brain tumors [15]. These results demonstrate that this genetic aberration is highly sensitive and specific to embryonal CNS tumors with multilayered rosettes irrespective of other features and that these subtypes are highly interrelated. Recently, Paulus and Kleihues therefore proposed to use the term “embryonal tumor with multilayered rosettes” (ETMR) as a general name for these tumors, a new entity, in part defined by the *C19MC* amplification itself [22].

To further test whether the three histological variants of ETMR represent a single entity, we performed clinicopathological and molecular analyses in 97 ETMR samples initially designated as ETANTR, EBL, or MEPL.

## Materials and methods

Ninety-seven diagnostic specimens diagnosed histopathologically as either ETANTR, EBL, or MEPL were received for this study from various sources around the globe and collected during the last 5 years. Among these sources were Burdenko Neurosurgical Institute, Moscow, Russia; University of Bonn, Germany; Ludwig-Maximilians University, Munich, Germany; University of Münster, Germany; University of Tübingen, Germany; Università Sapienza, Rome, Italy; Necker Hospital, Paris, France; Academic Medical Center, Amsterdam, the Netherlands; University of Cambridge, Cambridge, UK; Institute of Neurology, Vienna, Austria; Hospital for Sick Children, Toronto, Canada; Memorial Sloan Kettering Cancer Center, New York, USA; and University of California, San Francisco, USA. A subset of these cases was previously published [15, 16].

All cases were routinely formalin fixed and paraffin embedded. For diagnostic purposes, routine histopathological examination and immunohistochemical (IHC) analyses were performed in the different institutions participating in this study. Further centralized evaluation of all H&E slides was performed in the Heidelberg University Department of Neuropathology. In all 97 cases, IHC analysis applying a LIN28A polyclonal antibody and FISH analysis for the 19q13.42 locus were performed as previously described [15, 16].

For samples for which sufficient DNA was available (*n* = 41), we analyzed copy number aberrations (CNAs) using data generated with Illumina Human Methylation 450 k BeadChip arrays as described previously [13, 26]. For the detection of amplifications, chromosomal gains and losses, automatic scoring was verified by manual assessment of the respective loci for each individual profile as previously described [26].

To evaluate the molecular specificity of potential ETMR subtypes, we performed comparative cluster analysis of 450 k profiles generated for 41 ETMR together with 110 other primary pediatric brain tumors including pilocytic astrocytoma (PA; *n* = 10), ependymoma (EPN; *n* = 10), glioblastoma grade IV (GBM; *n* = 40), atypical teratoid rhabdoid tumor (AT/RT; *n* = 10), and medulloblastoma (MB; *n* = 40). Eight normal cerebellum (CBM) samples were also included. The following criteria were applied to filter the data: removal of probes targeting the X and Y chromosomes (*n* = 11,551), removal of probes containing a single-nucleotide polymorphism (dbSNP132 Common) within five base pairs of and including the targeted CpG-site (*n* = 24,536), and probes not mapping uniquely to the human reference genome (hg19) allowing for one mismatch (*n* = 9,993). In total, 438,370 probes were kept for analysis. For unsupervised hierarchical clustering of 41 ETMR samples, we selected the 4,756 most variably methylated probes across the dataset (s.d. >0.25). Samples were clustered using 1-Pearson correlation coefficient as the distance measure and average linkage (x-axis). Methylation probes were reordered by hierarchical clustering using euclidean distance and average linkage (y-axis). The heatmap illustration of 41 ETMR samples and 118 other pediatric brain tumor and control samples was generated by separately determining the 2,500 most variably methylated probes between the medulloblastoma subgroups, the K27-, G34-, IDH-, and wt (not H3.3 or IDH mutated) GBM subgroups, the non-ETMR tumor samples, and across the whole dataset. Probes were only used once (*n* = 6,540). Methylation probes were reordered by hierarchical clustering using euclidean distance and average linkage (y-axis).

## Results

### Pathological examination of the ETMR cohort

**Fig. 1:**
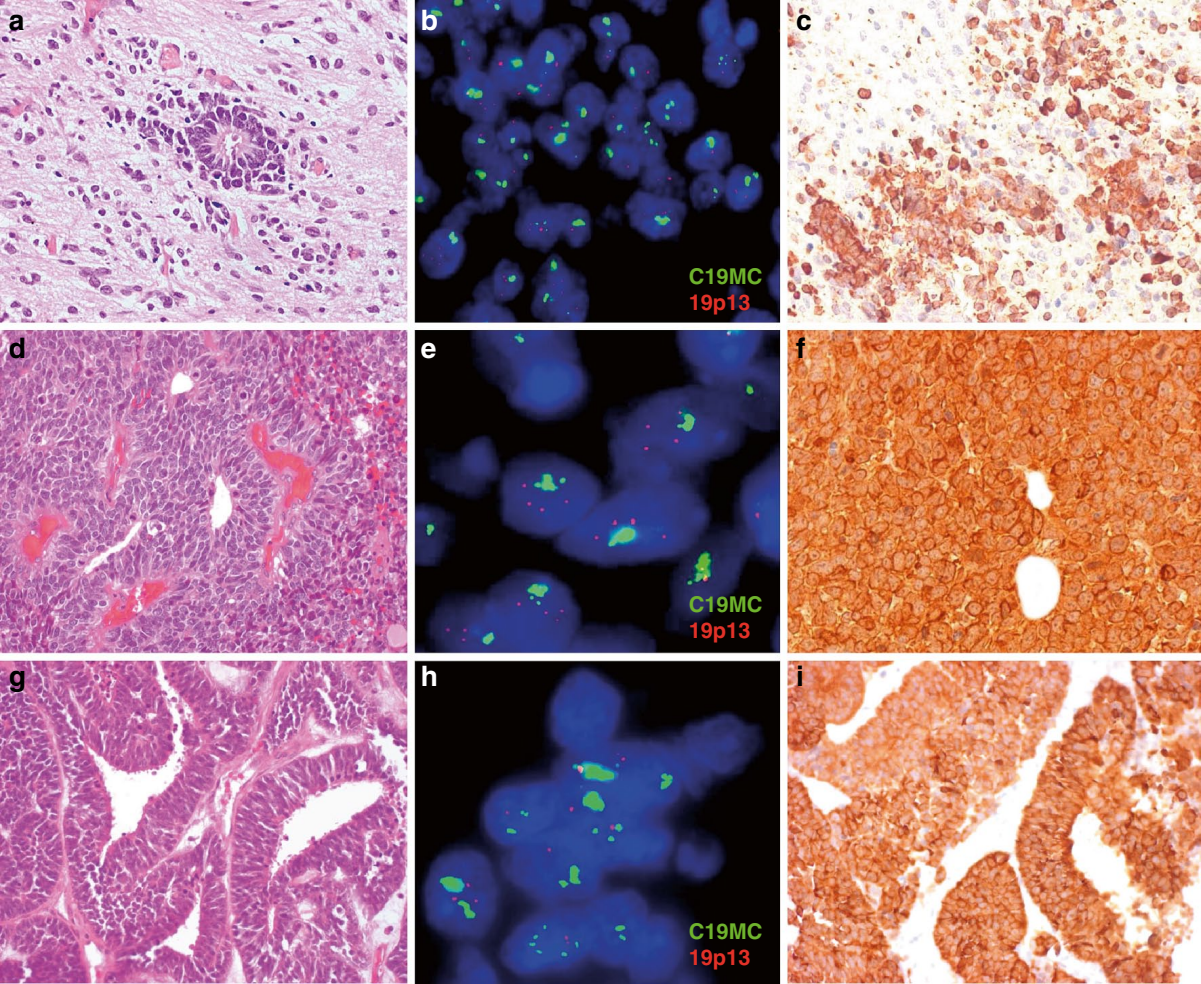
Microscopical appearance (**a**, **d**, **g**), FISH analysis of the 19q13.42 locus (**b**, **e**, **h**), LIN28A immunohistochemistry (**c**, **f**, **i**) of ETANTR (**a**–**c**), EBL (**d**–**f**) and MEPL (**g**–**i**). Amplification of 19q13.42 (**b**, **e**, **h**) and LIN28A immunoexpression (**c**, **f**, **i**) was detected in all three histological ETMR subtypes. For the FISH analysis the C19MC 19q13.42 probe (*green signals*) and a reference 19p13 probe were used (*red signals*)

According to published histopathological criteria, the 97 ETMR cases studied were diagnosed as ETANTR (55 cases), EBL (34 cases), or MEPL (eight cases) after central review. ETANTR was defined according to the criteria previously described by Eberhart et al. [6]. These tumors showed a biphasic pattern featuring highly cellular clusters of small cells with round or polygonal nuclei and scanty cytoplasm admixed with large fibrillar and paucicellular neuropil-like areas, infrequently containing neoplastic neurons. Among the clusters and aggregates of small cells, numerous true multilayered ependymoblastic rosettes were identified (Fig. 1a). In some cases, these rosettes were observed abruptly in the neuropil areas and neoplastic neurons were found between the cells composing layers of the rosettes. EBL and MEPL were diagnosed according to current WHO classification criteria [18]. EBL was identified as a tumor with the exclusive presence of nests and clusters of poorly differentiated cells forming true multilayered ependymoblastic rosettes but lacking a neuropil-like matrix (Fig. 1d). These rosettes were intermixed with small to medium-sized primitive cells having a high nucleus/cytoplasm ratio and variably developed fibrillary processes. MEPL was characterized by the presence of papillary, tubular, and/or trabecular arrangements of neoplastic pseudo-stratified epithelium with an outer membrane resembling the primitive neural tube (Fig. 1g). In zones distinct from tubular structures, large areas of poorly differentiated cells including collections of true multilayered ependymoblastic rosettes were found in all eight cases studied.

Recently, we have shown that expression of LIN28A is a highly specific and sensitive marker for pathological verification of ETMR [16, 23]. In the present series, strong and diffuse LIN28A cytoplasmic immunostaining was found in all 97 tumors studied irrespective of their histopathological appearance mentioned above (Fig. 1c, f, i). LIN28A positivity was found to be more prominent and intense in multilayered rosettes and poorly differentiated small-cell tumor areas of ETANTR/EBL, and in papillary and tubular structures of MEPL.

### Pathological analysis of recurrent ETMR samples

We were able to perform histological and molecular analysis of 14 samples obtained from local recurrences of ETMR: 11 were initially diagnosed as ETANTR, two as EBL, and one as MEPL. In addition, three samples from extracranial metastases of ETANTR (one in cranial soft tissue and two in the peritoneal cavity) were analyzed.

**Fig. 2:**
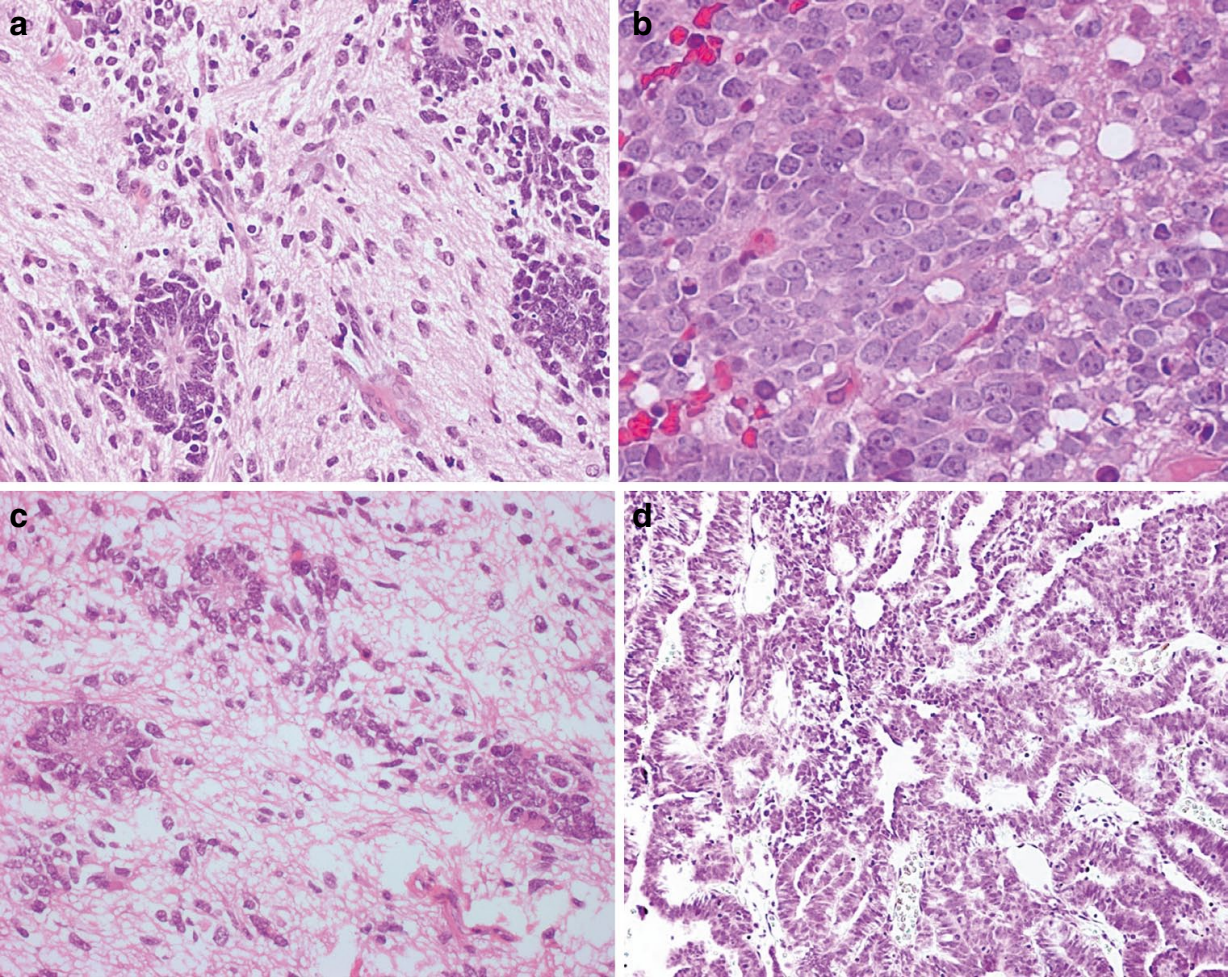
Two examples of primary ETANTR (**a**, **c**) with further tumor transformation in either EBL (**b**) or MEPL (**d**) histology as it has been identified during analysis of the recurrence samples

Upon secondary surgery at the time of relapse, histopathology in all 11 ETANTR samples showed a loss of neuropil-like foci. The secondary biopsy specimens obtained from nine ETANTRs disclosed extended collections of multilayered rosettes with various size and shape reflecting a histological pattern compatible with EBL (Fig. 2a, b). The other two samples showed prominent papillary and tubular structures, closely resembling MEPL (Fig. 2c, d). The extracranial metastasis of one ETANTR was predominantly composed of clusters of ependymoblastic rosettes varying in shape and size. In two other ETANTR metastases, papillary and tubular “MEPL-like” structures were detected. The two recurrent EBL samples and one MEPL showed histopathological features reminiscent of the corresponding primary tumors.

Immunohistochemical expression of the ETMR marker LIN28A was retained in all recurrent tumor samples and the number of positively stained cells in recurrent ETANTRs was higher in comparison to their primaries [16].

### Clinical parameters do not differ between the three ETMR groups

Basic clinical characteristics from all 97 patients included in this study are summarized in Table 1. All tumors occurred in very young children: range 0.5–6 years (median 2.3 years), and only eight patients (8 %) were older than 3 years. The male:female ratio was 1.1:1. The mean age for the three histological variants of ETMR did not differ significantly. Most tumors (64 cases; 70 %) were located supratentorially, with almost half of these (*n* = 30) found in the fronto-parietal region. Infratentorial location was less frequent (27 cases; 30 %) affecting either the cerebellum (*n* = 10) or brain-stem (*n* = 17). Exact information about tumor location was not available for six patients. Supra- and infratentorial location within the three ETMR groups did not differ significantly (Table 1). Data on metastatic stage at tumor diagnosis were available for only 32 patients. Most presented with M0 disease (26/32, 80 %), two with M2 stage, and four with M3 stage.

**Table 1:** Patient characteristics of 97 ETMR cases

Variable	ETANTR (*n* = 55)	EBL (*n* = 34)	MEPL (*n* = 8)
Age (years)			
Median	2.5	2.6	2.2
Range	1–5	0.5–6	1–3
Gender			
Male	29	15	3
Female	26	19	5
Tumor location			
Supratentorial	37	21	6
Infratentorial	14	11	2
Events			
Recurrence	29/32	16/17	5/6
Death	26/32	14/17	5/6
Survival			
Median PFS (months)	9.3	7.1	7.3
Median OS (months)	13.1	11.4	10.8

**Fig. 3:**
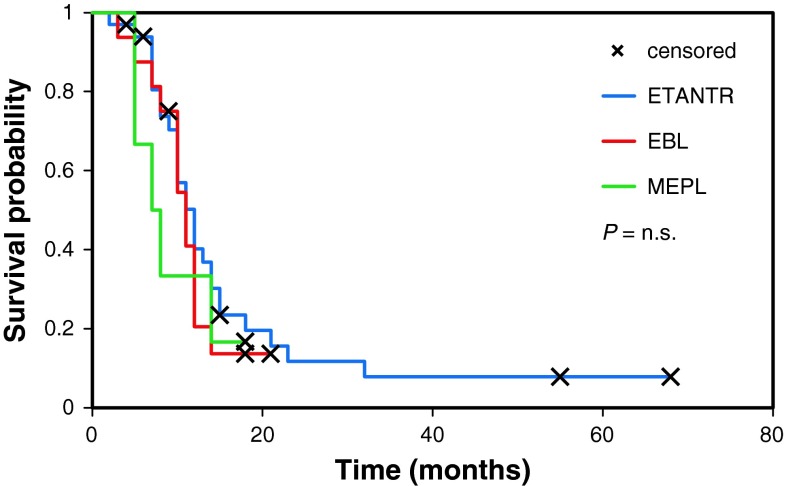
Overall survival curves generated for ETANTR (32 cases, *blue*), EBL (17 cases, *red*), and MEPL (6 cases, *green*). No differences in survival time were found (log-rank, *p* = 0.63)

Details of patient treatment were known for 36 patients (18 ETANTR; 14 EBL, and 4 MEPL). Sixteen patients had undergone gross total tumor resection, while for the other 20 children only subtotal tumor removal could be achieved. Only three patients received postoperative cranio-spinal irradiation, whereas all 36 patients were treated with chemotherapy based on the HIT-SKK2000 protocol [9]. Follow-up data were available for 55 patients demonstrating that 50 tumors recurred during follow-up with a median progression-free survival (PFS) of 8 months, and 84 % (46/55) of patients died within 3 years after their initial diagnosis. Median overall survival (OS) was 12.3 months and did not differ significantly between the three histological variants of ETMR (log-rank; *p* = 0.63, Fig. 3). Only two patients with an initial histological diagnosis of ETANTR are still alive more than 4 years after the first intervention (57 and 68 months, respectively).

Patterns of disease progression were highly variable. Most of the patients experienced local tumor regrowth as a first recurrence pattern, whereas a smaller number developed widespread leptomeningeal metastatic dissemination, very often after intervention on the recurrent lesion. Four patients additionally showed systemic metastases outside of the CNS in the cranial soft tissue, as well as pleural and peritoneal cavities.

### Cytogenetic analysis of 19q13.42 locus in ETMR

All 97 tumor samples were analyzed by FISH for amplification of the 19q13.42 locus. A high-level focal amplification of this locus (present in 40–80 % of tumor nuclei) was detected in 93 samples (96 %) (Fig. 1b, e, h). The four remaining tumors (two diagnosed as ETANTR and two as EBL) displayed polysomy 19, i.e., a low-level gain of both the target locus and the reference locus, compatible with an aberration affecting large parts of the chromosome or with an overall state of polyploidy. FISH analysis of the 14 recurrent tumors showed that the 19q13.42 amplification was retained in all samples. Moreover, the number of nuclei with amplification was significantly higher in secondary lesions (up to 100 % of nuclei) in comparison to their matched primary tumors [15].

### DNA copy number analysis and methylation profiling of ETMR

**Fig. 4:**
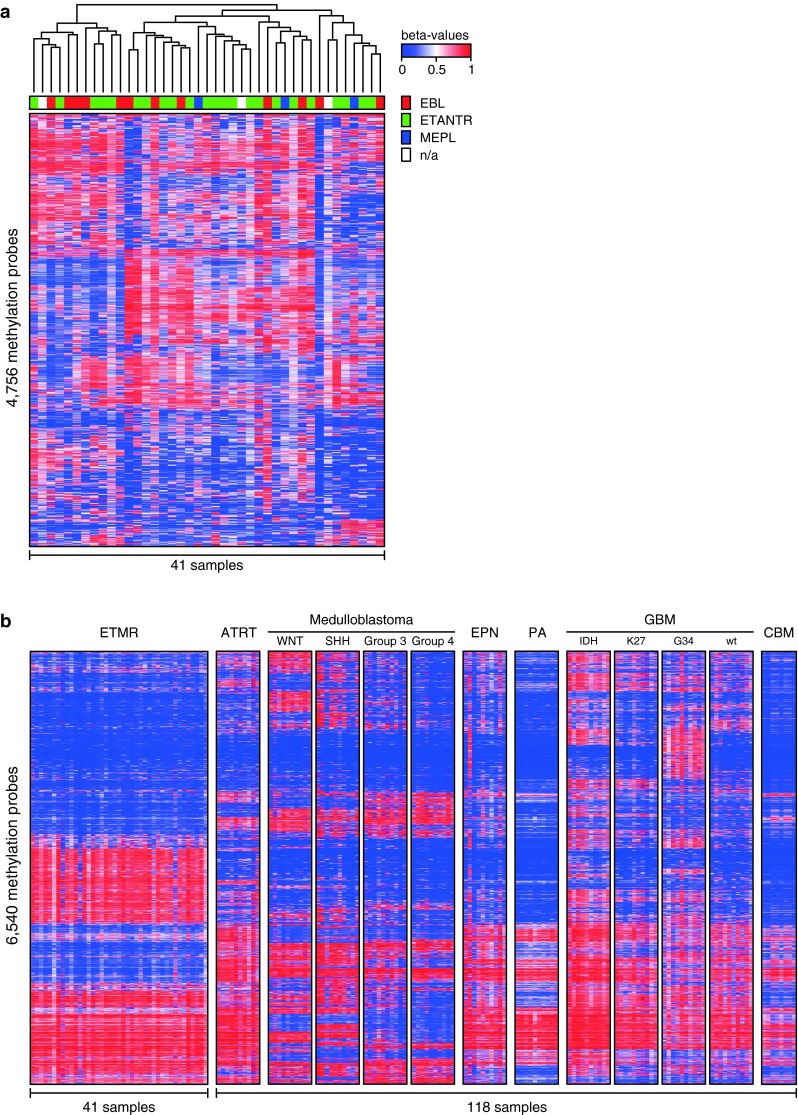
Cluster analyses of DNA methylation profiles of ETMR alone and compared to various other pediatric brain tumors and normal cerebellum. **a** Unsupervised cluster analysis of ETMR samples only shows that DNA methylation profiles of the histological variants ETANTR, EBL and MEPL are not distinct. Also, clusters outlined do not differ in terms of clinical findings, including age, gender, tumor location and outcome. **b** DNA methylation profiles of ETMRs are distinct from other pediatric brain tumors and normal cerebellum

**Fig. 5:**
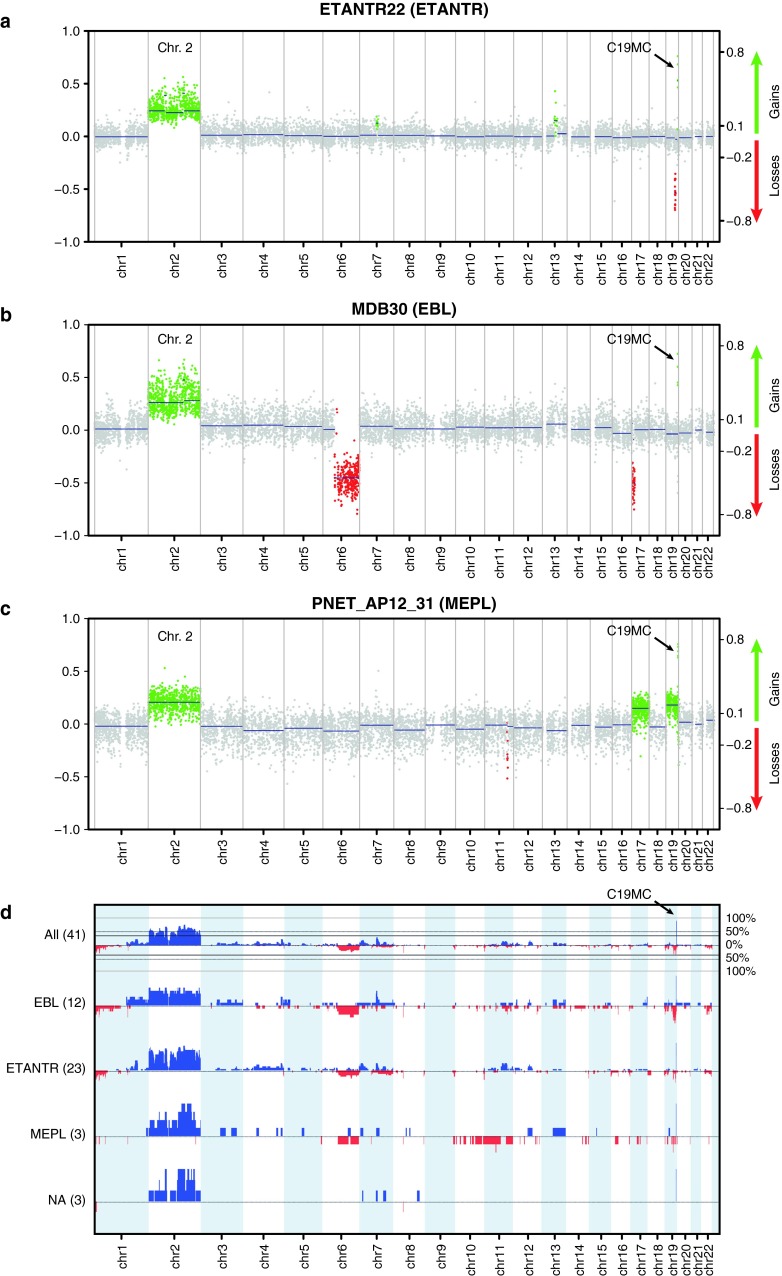
Copy number plots generated from 450 k methylation data. Amplifications and gains are indicated in* green*, losses in *red*. **a** Example of an ETANTR showing amplification 19q13.42, gain of 2 and loss of 19q13.3. **b** Example of an EBL showing amplification 19q13.42, gain of 2, and losses of 6q and 17p. **c** Example of a MEPL showing amplification 19q13.42, trisomy of 2, 17 and 19. **d** Summarizing profiles for all 41 cases analyzed

Next, we analyzed the genome-wide DNA methylation profiles of 41 ETMR samples using the Illumina 450 k DNA methylation arrays. As shown previously for glioblastoma and medulloblastoma [13, 26], DNA methylation profiling provides an excellent tool for the molecular sub-classification of distinct tumor entities. Unsupervised clustering analysis of the methylation data of the 41 ETMR samples did not reveal subgrouping according to ETMR histological subtypes (Fig. 4a). However, comparison to a large cohort of other pediatric brain tumors (*n* = 110) revealed that ETMRs are clearly distinct from other CNS tumors (Fig. 4b). In addition, data generated from these arrays can be used to detect CNAs in individual tumor samples. All CNAs detected from the methylation arrays for these 23 ETANTR, 12 EBL, 3 MEPL, and 3 additional histologically unidentified cases are outlined in Table 2. Amplification of the 19q13.42 locus was confirmed for 39/41 (95 %) primary tumors examined. Only one ETANTR and one EBL showed no amplification confirming the FISH analyses in these two samples. No other genomic amplifications were detected in this series of primary ETMR. Other recurrent low-level CNAs in these tumors included gain of chromosome 2 in 31 cases (76 %), gain of 7q (12/41, 29 %), gain of 11q (9/41, 22 %), gain of 1q (8/41, 20 %), and loss of 6q in eight cases (20 %). In eight cases, we additionally identified a focal genomic loss at 19q13.2–13.3, i.e., centromeric to the *C19MC* amplified region, suggesting complex intra-chromosomal rearrangements on the 19q13 locus in a subset of ETMR. No significant differences in the frequency of any of these CNAs were detected between the three histological ETMR variants (Fig. 5).

**Table 2:** Copy number aberrations in 41 ETMR cases

*N*	Age (years)	Gender	Location	Histology	Amplification	Gains	Losses	Outcome
1	2	F	Supra	ETANTR	19ql3.42	2	No	DOD (21 m)
2	2	M	Supra	ETANTR	19ql3.42	7	6q	NED (57 m)
3	3	F	Supra	ETANTR	19ql3.42	2	19q	DOD (18 m)
4	2	M	Infra	ETANTR	19ql3.42	2	No	DOD (l0 m)
5	1	F	Infra	ETANTR	No	lq, 19p	No	DOD (32 m)
6	2	M	Supra	ETANTR	19ql3.42	2, 14, 17, 20	No	D0D (12 m)
7	3	F	Supra	ETANTR	19ql3.42	2	No	DOD (15 m)
8	2	M	Supra	ETANTR	19ql3.42	2	No	DOD (10 m)
9	2	F	Infra	ETANTR	19ql3.42	7, llq	6q, 9, 12q, 16	DOD (23 m)
10	3	F	Supra	ETANTR	19ql3.42	2, 7, 11	6q, 19q	DOD (10 m)
11	3	M	Infra	ETANTR	19ql3.42	2, 4, 7, llq	lp, 19q	DOD (10 m)
12	2	F	Supra	ETANTR	19ql3.42	2, 21q	No	DOD (12 m)
13	3	M	Supra	ETANTR	19ql3.42	2, 16	No	DOD (11 m)
14	2	F	Supra	ETANTR	19ql3.42	No	6q	DOD (12 m)
15	3	M	Supra	ETANTR	19ql3.42	2	No	POD (6 m)
16	2	F	Supra	ETANTR	19ql3.42	2	No	NA
17	3	M	Supra	ETANTR	19ql3.42	2	No	DOD (14 m)
18	3	F	Supra	ETANTR	19ql3.42	No	7q	NA
19	2	M	Supra	ETANTR	19ql3.42	lq, 2, 4, 7q, 12q, 13q, 21q	8q, l0q	POD (4 m)
20	5	M	Infra	ETANTR	19ql3.42	2, 11	No	DOD (9 m)
21	2	M	Supra	ETANTR	19ql3.42	lq, 2, 6p, 7, llq, 21q	lp, lip, 18p, 22q	DOD (7 m)
22	3	F	Supra	ETANTR	19ql3.42	lq, 2, 4, 7, 8, 11, 16, 17, 20, 21q	No	NED (68 m)
23	2	M	Supra	ETANTR	19ql3.42	2, 15q	No	DOD (9 m)
24	2	F	Supra	EBL	19ql3.42	2, 3, 4, 7, 8, 13q	No	DOD (10 m)
25	2	F	Supra	EBL	19ql3.42	llq	lp, 19q, 22q	DOD (12 m)
26	3	M	Supra	EBL	19ql3.42	lq, 7	6q, 19q	DOD (10 m)
27	2	M	Supra	EBL	19ql3.42	6p, 7	No	DOD (10 m)
28	2	F	Supra	EBL	19ql3.42	lq, 2, 3q, 4	No	DOD (11 m)
29	2	M	Supra	EBL	19ql3.42	2	6q, 17p	DOD (6 m)
30	2	M	Infra	EBL	No	lq, 2, 17q	No	DOD (9 m)
31	3	F	Supra	EBL	19ql3.42	2	No	NED (6 m)
32	3	F	Infra	EBL	19ql3.42	2, 19, 20, 21q	No	NA
33	2	F	Supra	EBL	19ql3.42	2, 5q, 7, llq, 15q, 21q	22q	POD (11)
34	3	M	Supra	EBL	19ql3.42	lq, 2, 6p, 17, 20q	6q, 14q, 19q	POD (21 m)
35	2	F	Supra	EBL	19ql3.42	No	19q	NA
36	3	M	Supra	MEPL	19ql3.42	2, 5p, llq, 17q, 20q	lp, 6q, 12p, 19q	DOD (6 m)
37	2	M	Supra	MEPL	19ql3.42	2, 17, 19	No	DOD (5 m)
38	1	F	Supra	MEPL	19ql3.42	2	No	DOD (7 m)
39	NA	NA	NA	NA	19ql3.42	2	No	NA
40	NA	NA	NA	NA	19ql3.42	2	No	NA
41	NA	NA	NA	NA	19ql3.42	2, 7q, 8	No	NA

*ETANTR* embryonal tumor with abundant neuropil and true rosettes, *EBL* ependymoblastoma, *MEPL* medulloepithelioma, *DOD* died of disease, *POD* alive, progression of disease, *NED* alive, no evidence of disease, *NA* not available

## Discussion

Currently, the verification of a distinct nosologic position for various human malignancies is complemented (or sometimes defined) by a comprehensive molecular work-up [23, 26]. A number of refinements have recently been introduced into the current histological classification of pediatric CNS tumors. For example, the routine application of molecular diagnostics distinguishes AT/RTs from other PNETs [18] and current studies strongly suggest incorporating four molecular medulloblastoma subgroups into the classification as separate tumor entities [20]. These molecular data will help to further subdivide existing tumor entities by identifying disease variants with diverse clinical outcomes, distinct biology and clues regarding cell of origin. In contrast, molecular analyses of a representative cohort of ETANTR, EBL, and MEPL, three rare variants of embryonal CNS neoplasms, strongly suggests their integration into one single tumor entity called ETMR. All tumors were positive for LIN28A, a marker highly specific for ETMRs, and almost all cases displayed amplification of the *C19MC* miRNA cluster at 19q13.42, as well as frequent trisomy 2. Furthermore, no significant differences in other CNAs were observed between these three histological variants and their DNA methylation patterns were highly concordant.

Morphologically, ETMR manifests uniformly with the presence of multilayered true rosettes: “ependymoblastic” and/or “medulloepithelial”, with variable shape and size. As already noted, it may frequently be difficult to distinguish between EBL and MEPL [18]. Diagnostic differences between these tumor categories are descriptive and conceptual because they are, in general, based only on the absence of an outer collagen IV-positive membrane and apical cytoplasmic blebs in EBL structures. Moreover, similar clinical parameters and a highly aggressive course of disease for all ETMR histological variants (resistance to treatment, inevitable tumor recurrence and rapid death) also support our suggestion of commonality [1, 8, 9, 11, 15, 16, 19].

Previously, molecular analysis of various ETMR subtypes has been hampered by limited cohort sizes and to date, only a few chromosomal imbalances were detected by conventional CGH analysis [7, 25]. Recently, we described a focal unique amplicon at 19q13.42 spanning 0.89 Mb and covering the *C19MC* genomic locus, the largest known cluster of human microRNA-coding genes [24]. Further, the *C19MC* locus was found to be amplified in 93 % of ETMR diagnosed previously on the basis of their characteristic morphology [15], and these findings have been confirmed by others [5, 17, 21, 23, 27, 28]. Thus, *C19MC* amplification is a highly specific genetic ETMR hallmark, similar to loss of the *SMARCB1* locus in AT/RT or t(11;22) in Ewing sarcoma [18]. This suggests that the *C19MC* amplification could serve as a “driver” oncogenic event in ETMR. Comprehensive analysis of CNAs in our ETMR cohort revealed few other recurrent chromosomal gains and losses, and no subtle cytogenetic differences between the tumors with various histological features could be defined.

Common molecular signatures between the three histological variants of ETMR suggest that they may share a common origin, such as a primitive cell population in the subependymal region, with further evolution into a wide range of morphological appearances and mimics. In support of this suggestion, analysis of the 11 recurrent ETANTR samples revealed its frequent histological evolution into either EBL or MEPL phenotypes, while at the same time the molecular genetic make-up did not change between the initial and advanced stages of disease. Such stepwise morphologic transformations in the “classic” ETANTR appearance due to disease progression allow one to suppose that the three “histological variants” of ETMR may constitute either “polar ends” of a morphologic spectrum or varying differentiation stages of a single tumor entity rather than separate nosologic categories.

In conclusion, we identified uniform molecular signatures occurring in a representative subset of embryonal brain tumors with multilayered rosettes indicating that ETANTR, EBL, and MEPL comprise a single biological entity, which could potentially be designated in future WHO schemes as ETMR. For molecular diagnosis of this tumor category and to distinguish them from other embryonal tumors of the CNS, combined LIN28A IHC and FISH analysis of the 19q13.42 locus are recommended as routine diagnostic markers. Since misdiagnosis for controversial “poorly differentiated” embryonal CNS neoplasms is not uncommon, all tumors and especially CNS-PNETs harboring combined LIN28 expression and 19q13.42 amplification should be classified as ETMR, even in absence of the key histological patterns. As a next step, it will be important to understand the distinct biological significance of the prototypic molecular events in ETMR which may provide therapeutic targets for novel treatment strategies for these highly aggressive and therapy-resistant pediatric CNS malignances.
